# Chronic inflammatory injury results in increased coupling of delta opioid receptors to voltage-gated Ca^2+^ channels

**DOI:** 10.1186/1744-8069-9-8

**Published:** 2013-03-04

**Authors:** Amynah Pradhan, Monique Smith, Brenna McGuire, Christopher Evans, Wendy Walwyn

**Affiliations:** 1 Department of Neuropsychiatry and Biobehavioral Sciences, Stefan and Shirley Hatos Center for Neuropharmacology, Semel Institute, University of California, Los Angeles, CA, 90095, USA

**Keywords:** Primary afferent, SNC80, Delta opioid receptor, Chronic pain, Dorsal root ganglia, Ca^2+^ channel

## Abstract

**Background:**

Opioid receptors regulate a diverse array of physiological functions. Mu opioid receptor agonists are well-known analgesics for treating acute pain. In contrast, animal models suggest that chronic pain is more effectively relieved by delta opioid receptor agonists. A number of studies have shown that chronic pain results in increased function of delta opioid receptors. This is proposed to result from enhanced trafficking of the delta opioid receptor to the cell membrane induced by persistent tissue injury. However, recent studies have questioned this mechanism, which has resulted in some uncertainty as to whether delta opioid receptors are indeed upregulated in chronic pain states. To clarify this question, we have examined the effect of chronic inflammatory pain over time using both an *ex vivo* measure of delta function: receptor-Ca^2+^ channel coupling, and an *in vivo* measure; the relief of chronic pain by a delta opioid receptor agonist. In addition, as beta-arrestin 2 can regulate delta opioid receptor trafficking and signaling, we have further examined whether deleting this scaffolding and signal transduction molecule alters delta opioid receptor function.

**Results:**

We used the Complete Freund’s Adjuvant model of inflammatory pain, and examined the effectiveness of the delta agonist, SNC80, to both inhibit Ca^2+^ channels in primary afferent neurons and to attenuate mechanical allodynia. In naïve beta-arrestin 2 wildtype and knockout mice, SNC80 neither significantly inhibited voltage-dependent Ca^2+^ currents nor produced antinociception. However, following inflammatory pain, both measures showed a significant and long-lasting enhancement of delta opioid receptor function that persisted for up to 14 days post-injury regardless of genotype. Furthermore, although this pain model did not alter Ca^2+^ current density, the contribution of N-type Ca^2+^ channels to the total current appeared to be regulated by the presence of beta-arrestin 2.

**Conclusions:**

Our results indicate that there is an upregulation of delta opioid receptor function following chronic pain. This gain of function is reflected in the increased efficacy of a delta agonist in both behavioral and electrophysiological measures. Overall, this work confirms that delta opioid receptors can be enhanced following tissue injury associated with chronic pain.

## Background

Opioid receptors regulate diverse physiological processes, including reward, pain, and stress (see [1,2]). The mu opioid receptor (μOR) is the best characterized member of this family, and μOR agonists are some of the most clinically effective analgesics. However, there are a number of severe drawbacks with the use of μOR agonists such as respiratory depression, sedation, and constipation. Importantly, μOR agonists are also extremely addictive, as shown by the high abuse rates of the pharmaceutical opiates vicodin and oxycodone [3].

Drugs that activate the delta opioid receptors (δORs) do not result in these severe μOR-associated side-effects and so could offer a promising alternative for treatment of certain types of pain [4]. Although δOR agonists are not highly efficacious in relieving acute pain, selective activation of these receptors has been shown to relieve chronic inflammatory [5-8] and neuropathic [5,9,10] pain in rodent models. These observations suggest that chronic pain is associated with a functional upregulation of δORs, proposed to be due to enhanced trafficking of δORs to the cell membrane. Electron microscopy studies propose that δORs are, for the most part, found in the sub-plasmalemmal space and that tissue injury relocates these receptors to the cell membrane [6,11-18]. However, the specificity of the antibodies used to label δORs has recently been questioned ([19,20] and see [21,22]). In addition, mice expressing δORs fluorescently tagged with enhanced Green Fluorescent Protein (DOR-eGFP) in place of endogenous receptors indicate that δORs are normally found at the cell surface in the central and peripheral nervous systems [7,8,20,23]. These results suggest that increased trafficking of δORs to the cell membrane following a painful insult may not be solely responsible for the increased functionality of these receptors. This controversy is further complicated by the known differences in resolution of standard confocal fluorescence vs. electron microscopy, and the possibility that a C-terminus eGFP tag may alter the cellular localization of δORs [24]. These differences have led to some uncertainty as to how δORs are upregulated in chronic pain states.

Irrespective of the mechanism by which δOR function is altered in chronic pain, we have asked a fundamental question: does chronic pain induce a functional enhancement of δORs in dorsal root ganglia (DRG)? We have used two measures of δOR functionality; δOR inhibition of voltage-dependent Ca^2+^ currents (VDCCs) in acutely dissociated DRGs and the ability of SNC80 to relieve chronic pain, and compared the ability of a δOR agonist to alter these parameters in naive and chronic pain states. We focused on medium-large sized DRGs that have been shown to express the δOR and to modulate mechanical pain [20]. Furthermore, as β-arrestin 2 has been shown to play a key role in δOR agonist-induced receptor trafficking and function [25], we examined whether β-arrestin 2 alters these parameters following CFA.

## Results

### Chronic inflammatory pain does not alter voltage-dependent Ca^2+^ channel function in medium-large sized DRGs but does result in mechanical allodynia

We first characterized the effect of chronic inflammatory pain, induced by Complete Freund’s Adjuvant injected into the hindpaw, on VDCCs and on mechanical sensitivity. Medium-large sized DRG neurons of equal capacitance and therefore cell size (naive; 61 ± 5, CFA; 64 ± 6 pF), were assessed by the whole cell patch clamp technique under voltage clamp conditions. CFA did not alter the current–voltage relationship (Table 1). Furthermore, Ca^2+^ channel conductance, assessed from the maximal tail-currents from these current–voltage recordings, showed no effect of CFA on the conductance-voltage relationship (Table 1). There was also no effect of CFA on the steady state inactivation of Ca^2+^ currents (Table 1). Furthermore CFA did not alter constitutive, voltage-dependent current inhibition, (Table 1), or the ability of an ubiquitously expressed G_*i/o*_ GPCR, the GABA_B_ receptor, to inhibit VDCCs (Table 1). However, CFA induced a hypersensitivity to mechanical stimulation, as observed by a decrease in the 50% withdrawal threshold as measured with manual von Frey hair stimulation (naive: 0.99 ± 0.03, CFA: 0.17 ± 0.01, p < 0.001, F _(1,15)_ = 97.60). In summary, this model of chronic inflammatory pain did not alter the properties of voltage-dependent Ca^2+^ currents in medium-large DRG neurons but, as expected, resulted in mechanical hyperalgesia.

**Table 1:** **Voltage-dependent properties of Ca**^**2+ **^**channels in DRGs from naive and CFA-treated mice**

	**Naive**	**CFA**
**Activation**		
Gmax	308 ± 51	288 ± 41
Slope	6.1 ± 0.8	6.5 ± 0.9
V_1/2_	−25.2 ± 1.0	−25.6 ± 1.0
**Inactivation**		
Slope	16.5 ± 2.5	9.9 ± 0.9
V_1/2_	−13.2 ± 4.8	−14.0 ± 1.3
**Constitutive Inhibition**		
	1.00 ± 0.02	1.01 ± 0.02
**GABA**_**B **_**inhibition (%)**		
	42.4 ± 3.2	45.5 ± 4.3

Patch-clamp recordings under voltage-clamp conditions were used to examine Ca^2+^ channel function in medium to large-sized L4-L6 DRG neurons from naive (61± 5 pF) and CFA (64 ± 6 pF) treated mice. CFA did not alter the maximal current amplitude (G_max_; pA/pF; F_(13, 234)_ = 0.21), the kinetics of channel activation (p > 0.05, F_(13,126)_ = 0.04), steady-state inactivation ( p > 0.05, F_(12,2040)_ = 1.3), constitutive current inhibition (p > 0.05, t_14_ = 0.89) or GABA_B_ inhibition of the Ca^2+^ channels (p > 0.05, t_12_ = 0.59). V_1/2_ = half-maximal potential of the conductance-voltage relationship.

### Delta opioid receptors show minimal functionality under basal conditions

**Figure 1:**
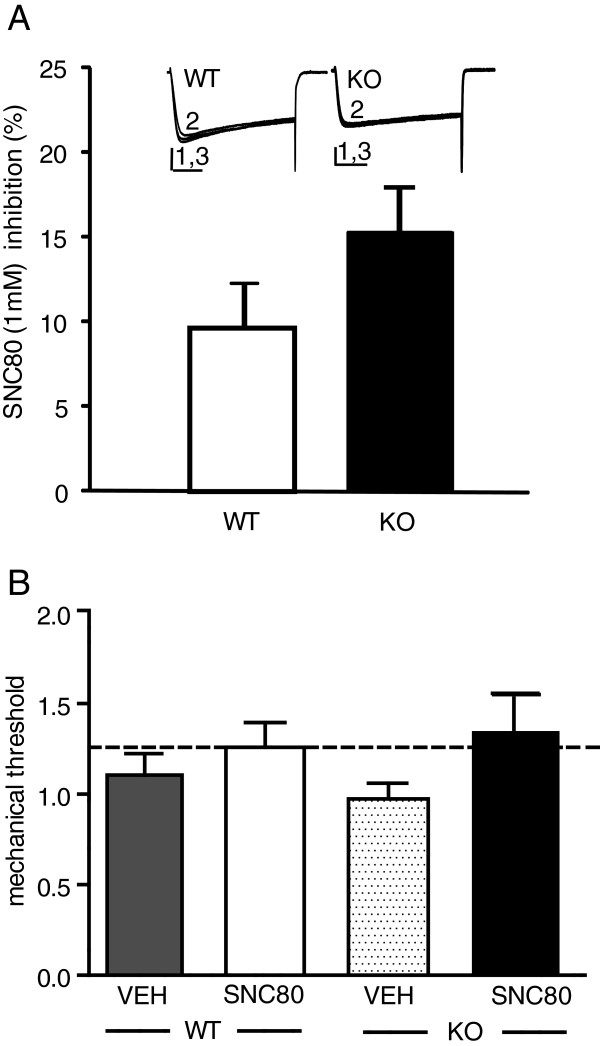
**In naïve pain-free mice, SNC80 shows minimal Ca**^**2+ **^**channel inhibition in DRG neurons and does not alter the response threshold to von Frey hair stimulation. A**. SNC80 applied to dissociated DRG neurons from adult mice showed minimal VDCC inhibition equally in β-arrestin 2 WT or KO mice (F_1,41_ = 1.66). Exemplar currents show VDCCs before (1), during (2) and after (3) SNC80 (1μM) application, vertical scalebar = 20 ms and horizontal scalebar = 0.5 pA, n = 20–22. **B**. β-arrestin 2 WT and KO mice were tested with vehicle or SNC80 (10 mg/kg, i.p.), and mechanical responses were assessed 45 min later. Baseline mechanical responses are represented by the dashed line. There was no significant effect of SNC80 in either genotype, as determined by 2-way ANOVA (n = 4-5mice/group).

We next examined δOR receptor function under basal conditions. As δORs are a member of the G_*i/o*_-coupled family of G-protein coupled receptors (GPCRs) and able to inhibit VDCCs in DRG neurons we assessed VDCC inhibition induced by SNC80, a specific δOR agonist. We found low levels of SNC80-VDCC inhibition, in medium-large sized DRG neurons from untreated mice (WT, 9.6 ± 2.8% and KO, 15.3 ± 2.7% , F_1,41_ = 1.66, Figure 1A). We also assessed whether SNC80 could alter the response to a mechanical stimulus in naïve mice but found no effect of SNC80 (Figure 1B). Together these parameters suggest that δORs are mostly quiescent under basal conditions.

### Chronic inflammatory pain results in an increased efficacy of SNC80 to inhibit VDCCs and to relieve chronic pain

**Figure 2:**
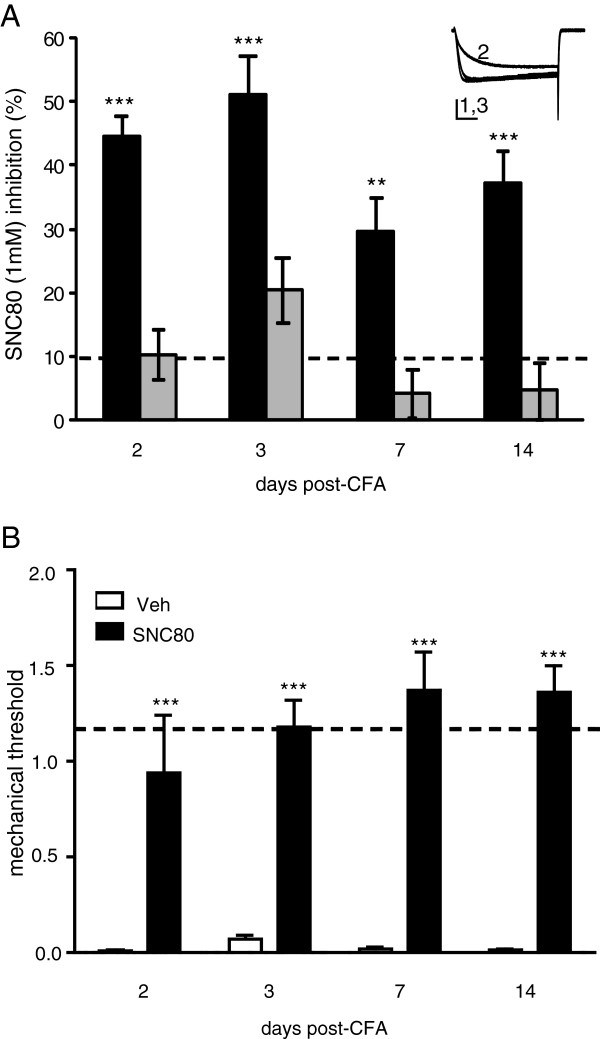
**In wildtype mice CFA increased SNC80-Ca**^**2+ **^**channel inhibition and reversed CFA-induced allodynia. A**. CFA induced an increase in SNC80-VDCC inhibition in WT DRG neurons from the ipsi-, but not contra-lateral sides to the CFA injection; ANOVA: F _(8,78)_ = 14.86, ** p < 0.01 and *** p < 0.001 vs. basal/naive inhibition, shown by the dashed line. An exemplar current shows VDCCs before (1), during (2) and after (3) SNC80 (1 μM) application, vertical scalebar = 20 ms and horizontal scalebar = 0.5 pA, n = 6–20. **B**. The effect of SNC80 (10 mg/kg i.p.) or saline was assessed in mice which had been treated with CFA 2, 3, 7 and 14 days prior to the test. Baseline mechanical responses are represented by the dashed line. *** p < 0.001 as determined by 2-way ANOVA with a Holm-Sidak post-hoc analysis (n = 4–8 mice/group).

We then examined whether CFA alters δOR function. We found an increase in δOR-VDCC inhibition above basal levels 2, 3, 7 and 14 days after the CFA injection in DRGs from the ipsi-, but not contra-lateral sides to the CFA injection in WT mice (Figure 2A). These data reflect both an increase in SNC80-VDCC inhibition and an increase in the number of cells responding to SNC80, as assessed by the percentage of cells in which SNC80 inhibited VDCCs by more than 10%. Using this criterion, 31% of the DRGs from the naive group responded to SNC80 compared to 100% of DRGs from all time points following CFA injury. Reflecting these *ex vivo* data, SNC80 significantly attenuated CFA-induced mechanical allodynia 2, 3, 7 and 14 days after CFA injection (Figure 2B).

### SNC80 inhibits VDCCs and relieves pain in CFA-treated β-arrestin 2 knockout mice

**Figure 3:**
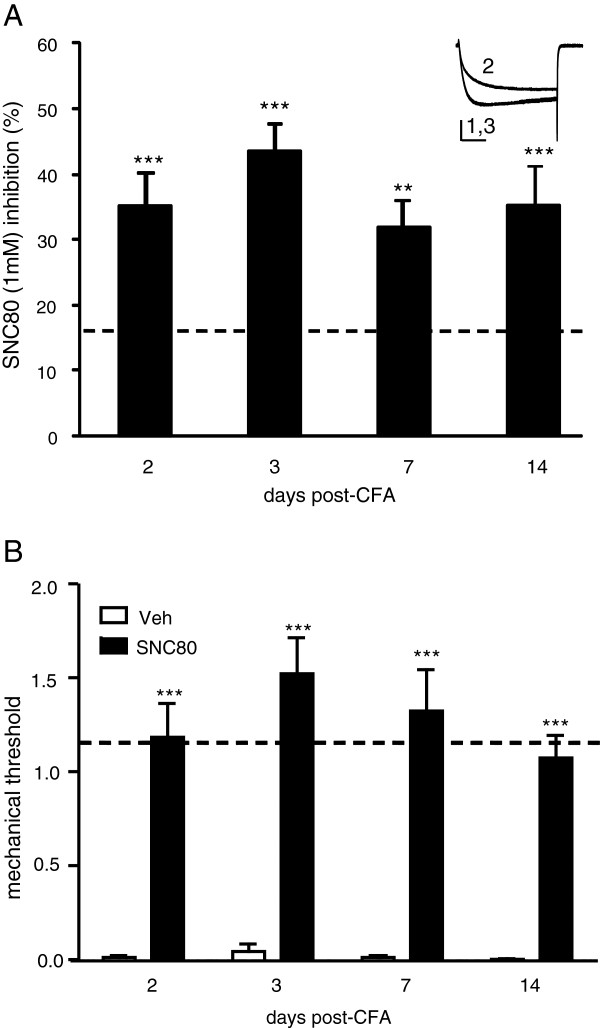
**In β-arrestin 2 knockout mice CFA increased SNC80-Ca**^**2+ **^**channel inhibition and reversed CFA-induced allodynia. A**. Although basal SNC80-VDCC inhibition tended to be higher in KO mice, CFA increased SNC80-VDCC inhibition in DRG neurons above basal levels; ANOVA: F_(4,58)_ = 9.83), ** p < 0.01 and *** p < 0.001 vs. basal/naive inhibition, shown by the dashed line. An exemplar current shows VDCCs before (1), during (2) and after (3) SNC8 (1 μM) application, vertical scalebar = 20 ms and horizontal scalebar = 0.5 pA, n = 6–14. **B**. The effect of SNC80 (10 mg/kg i.p.) or saline was assessed in mice which had been treated with CFA 2, 3, 7 and 14 days prior to the test. Baseline mechanical responses are represented by the dashed line. *** p < 0.001 as determined by 2-way ANOVA with a Holm-Sidak post-hoc analysis (n = 5–9 mice/group).

SNC80-VDCC inhibition demonstrated a similar effect in β-arrestin 2 knockout (KO) neurons, increasing above basal levels 2, 3, 7 and 14 days after CFA injection (F_(4,58)_ = 9.83, Figure 3). The number of neurons showing >10% response to SNC80 reflected these levels of inhibition, increasing from 63% in naive DRGs to 100% in DRGs taken from mice 3 days post-CFA. Similar to WT mice, SNC80 reversed CFA-induced mechanical allodynia 2, 3, 7 and 14 days after CFA injection in KO mice (Figure 3A).

### Deleting β-arrestin 2 does not alter current density but does reduce the contribution of N-type Ca^2+^ channels in β-arrestin 2 KO DRGs

**Figure 4:**
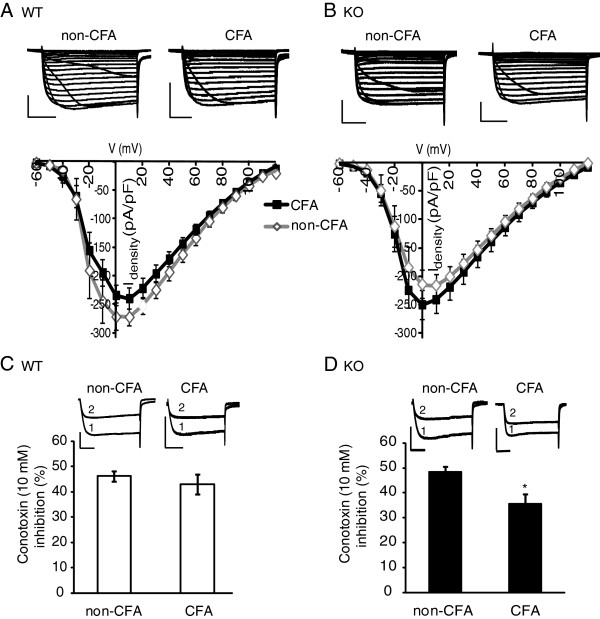
**Inflammatory pain altered VDCC-density and current-type contribution in β-arrestin 2 knockout, but not wildtype mice. A** and **B**. The current–voltage relationship recorded from either WT (**A**) or KO (**B**) neurons was not altered by CFA (3 days post-injection). Exemplar currents from WT and KO neurons of the contralateral (non-CFA) and ipsilateral (CFA) sides are shown above the current–voltage graphs depicting the maximum current induced by each voltage and corrected for cell capacitance. Vertical scalebar =20 ms and horizontal scalebar = 0.5 pA, n = 10–16. **C** and **D**. The contribution of N-type VDCCs to the total current in WT neurons was not altered by CFA (**C**) but was reduced in DRG neurons from KO mice (**D**; F _(3,25)_ = 4.57, n = 6–7). Exemplar currents show VDCCs measured in the absence (1) and presence (2) of the selective N-type inhibitor, *ϖ* -Conotoxin GV1A (10 μM). * p < 0.05.

Although we found no affect of CFA on Ca^2+^ current density in naive vs. CFA-treated medium-large size DRGs (Table 1), we further assessed the effect of genotype, KO vs WT, on current density. Supporting our previous findings (Table 1), we found no effect of CFA on the current density of DRGs from ipsi- vs. contra-leral sides. In addition, there was also no effect of genotype (Figure 4A and B). As N-type Ca^2+^ channels are the prevalent form of Ca^2+^ channels coupled to G_*i/o*_ GPCRs in DRG neurons [26], we then determined the contribution of N-type currents to the total current by assessing the effect of the N-type inhibitor, *ϖ* -Conotoxin GV1A (10 μM) on total current amplitude. In WT mice, the N-type contribution was equivalent in DRGs from both CFA and non-CFA sides (Figure 4C). However, DRGs from the CFA side of β-arrestin 2 KO mice showed less N-type contribution to the total current than seen in neurons from the non-CFA side (Figure 4D).

## Discussion

These data demonstrate that inflammatory pain induced by CFA results in increased functionality of δORs in DRG neurons. This is shown by an increased inhibition of Ca^2+^ channels by the δOR agonist, SNC80, which mirrored an enhanced efficacy of SNC80 to inhibit mechanical allodynia. These data indicate that, regardless of the trafficking events that may or may not be involved, chronic inflammatory pain produces an enhanced responsivity of δORs.

In this study, we used δOR-VDCC coupling in DRGs as an *ex vivo* measure of δOR function that correlates with the pain-relieving effects of δOR agonists. We found that in a naïve, injury-free state the δOR agonist SNC80 did not alter the response threshold to von Frey filaments. However, following induction of inflammatory pain SNC80 potently inhibited CFA-induced allodynia. This *in vivo* gain of function was mirrored by an increased efficacy of SNC80 to inhibit Ca^2+^ channels within the DRGs. These results reflect previous work which has shown that compared to μ agonists, δ agonists are poor analgesics in acute pain [27] yet, they are highly effective in chronic inflammatory and neuropathic pain, likely due to an induction of δ receptor function following chronic pain [6,28-31]. Further, the pain-relieving effects of δOR agonists has been previously shown to be mediated at the level of primary afferent neurons [10], supporting the notion that changes in Ca^2+^ channel coupling within the primary afferents would reflect behavioral responding. In addition, we had also shown a similar correlation following chronic use of δOR agonists, where uncoupling of δORs from VDCCs were observed following analgesic tolerance [8]. However, as δOR-VDCC coupling in DRGs is one of several pathways activated by δOR agonists [32-34], it is likely that the analgesic effects of δOR agonists reflect the co-operative influence of these different signaling cascades that may include δOR inhibition of Ca^2+^ channels.

δORs are a member of the G_*i/o*_-coupled family of G-protein coupled receptors (GPCRs) and, although able to inhibit VDCCs in DRG neurons, δOR agonists have not been shown to produce significant VDCC inhibition in the basal state (Figure one, [35]). Chronic inflammatory pain increased δOR-VDCC inhibition which could have been a result of several factors. Likely candidates include; an increase in the number of receptor-complexes available for ligand activation; changes in the number or kinetics of Ca^2+^ channel recruitment by these activated receptors; or an altered signaling pathway by which δORs inhibit VDCCs. CFA has been shown previously to reduce Ca^2+^ channel density in small to medium sized (<40 μM) DRG neurons [36]. However, we did not observe any effect of CFA on the voltage-dependent properties of Ca^2+^ currents in medium to large-sized DRG neurons. It is also unlikely that CFA induced an increase in receptor transcript and protein levels as neither have been reported to occur previously [18,37]. However, as δORs have been found as signalosomes associated with their cognate G-proteins and other signaling molecules [38], it is possible that CFA altered the composition of these signalosomes. This may be in addition to, or independent of, an increase in the number of receptors on the cell membrane as previously suggested [6,18,39,40]. Interestingly other paradigms such as treatment with bradykinin, chronic morphine, hypoxia and alcohol have also been shown to increase δOR function [33,41-46] suggesting that δOR upregulation may have a number of clinically useful roles [47].

Internalized δORs are primarily targeted for degradation [7,48-51] but some receptors may also be recycled [52] through the slow recycling, Rab11-dependent pathway [53]. Several lines of evidence indicate that β-arrestin 2 mediates this trafficking of δORs following receptor internalization [25,52] suggesting that we may have observed an altered response in β-arrestin 2 KOs. However, we found no effect of deleting β-arrestin 2 on the analgesic profile of SNC80, or on the enhanced δOR-VDCC coupling or VDCC density following CFA. However, we did observe that β-arrestin 2 plays a role in the contribution of N-type Ca^2+^ channels to the total Ca^2+^ current following CFA. Of the different types of VDCCs that contribute to the high voltage-activated currents in DRGs, the N-type normally contributes ~50% of the current [54]. In KO neurons, this decreased to ~35% suggesting an increase in the contribution of R or P/Q type channels so as to maintain total current density. This raises an intriguing possibility that β-arrestins may regulate the contribution of Ca^2+^ channels to the total current following CFA.

## Conclusions

In summary, our results indicate that chronic inflammatory pain results in an enhancement of δOR function, both at the level of behavioral responding and at the level of Ca^2+^ channel coupling in dorsal root ganglia neurons. This increased functionality may be due to changes in receptor trafficking or differences in receptor-effector complexes already at the cell membrane. This study shows that δ opioid receptors are responsive following tissue injury, and may become a promising target for the treatment of chronic pain.

## Methods

### Animals

β-arrestin 2 mutant mice were generously provided by Dr. Lefkowitz (Duke University). β-arrestin 2 (KO) and wild-type (WT) mice for both electrophysiology and behavioral experiments were obtained through heterozygous pairings. Both male and female mice were used between 8–24 weeks of age. All animal experiments were conducted in accordance with the AALAC Guide for the Care and Use of Laboratory Animals and followed institutionally approved animal care and use protocols; OARO: 2010-025-03B and 1999-179-41.

### DRG preparation

Delta receptor inhibition of VDCCs was assessed in acutely dissociated L4-L6 DRGs from untreated adult mice or mice that had undergone Complete Freund’s Adjuvant injection to induce chronic inflammation in the left paw. The DRGs were collected in Complete Saline Solution (CSS; in mM, NaCl: 137, KCl: 5.3, MgCl_2_:1, Sorbitol: 25, HEPES: 10, CaCl_2_: 3) and incubated in collagenase (1.25U of TH, Roche, Indianopolis, IN), 250 nm EDTA for 20 min at 32 C, transferred to fresh CSS containing collagenase (1.25U of TM, Roche) with 250 nm EDTA and 0.25U papain (Roche) and incubated for 10 min at 32 C. After 2 washes and physical trituration through a series of graded Pasteur pipettes, the cells were spun (1000 rpm, 3 min) and plated in Neurobasal /B27/Glumax/Antibiotic/Antimycotic (Life Technologies, Grand Island, NY) supplemented with 10 ng/ml NGF (Life Technologies). All recordings were performed within 5–24 h after plating.

### Electrophysiology

VDCCs were recorded from medium-large sized DRG neurons (30–100 pF) under whole-cell voltage-clamp conditions as previously described [54,55]. The cells were perfused with an external solution containing 10 mM CaCl_2_, 130 mM tetraethylammonium chloride, 5 mM HEPES, 25 mM *d*-glucose and 0.2 μM tetrodotoxin at pH 7.35 (Sigma). The patch electrode was filled with an internal solution composed of 105 mM CsCl, 40 mM HEPES, 5 mM *d*-glucose, 2.5 mM MgCl_2_, 10 mM EGTA, 2 mM Mg-ATP and 0.5 mM GTP at pH 7.2 (Sigma). Episodic recordings were obtained using an Axopatch 200B patch-clamp amplifier set at a gain of 1.0, β = 0.1 and 2 kHz filter. Capacitance and series resistance were corrected and series resistance compensated by 80 to 90% and included a 10 μs lag. Leak currents were subtracted using a P/6 protocol. Recorded signals were acquired and analyzed using Axon pCLAMP v9 or 10 software (Axon Instruments, Foster City, CA).

### The properties of voltage-dependent Ca^2+^ currents

Ca^2+^ currents were evoked every 20 sec by 100 ms voltage steps from −80 to +10 mV. Ca^2+^ channel density and conductance was assessed by evoking Ca^2+^ currents from −100 to + 40 mV in 10 mV increments with a 500 ms hyperpolarizing pre-pulse to 120 mV. Steady state inactivation was assessed by a test voltage pulse from −80 to +10 mV preceded by pulses of increasing voltage from −120 mV to +10 mV in 10 mV increments. The presence of constitutively coupled channels was measured by a 2-pulse protocol in which a 40 ms depolarizing pre-pulse from −120 to +40 mV preceded the 40 ms test pulse from −80 to +10 mV.

#### Statistical analysis

Ca^2+^ channel conductance from individual cells was fitted with the Boltzman equation; G/G_max_ = [1 + exp(V-V_1/2_/slope)^-1^ where G is the conductance of the test pulse, G_max_ is the maximal conductance, V is the voltage of the test pulse and V_1/2_ is the potential corresponding to the half-activation of the current. A modified Boltzman equation was used to assess steady-state inactivation; I/I_max_ = [1 + exp(V_1/2_-V/slope)^-1^ where I is the peak current of the test pulse and I_max_ is the maximal current [56]. Further analysis between groups was assessed by two-way ANOVA with repeated measures (Prism v5.0). Constitutive activity was determined by comparing the amplitude of the test pulse in the absence (P1) or presence of the pre-pulse (P2), expressed as a ratio (P1/P2) and analyzed by the Student’s *t*-Test (Prism v5.0).

### Drug application

Once 3–4 stable recordings were obtained, external solution containing SNC80 1 μM, R&D, Minneapolis, MN) or Baclofen (50 μM, Sigma) was applied to the cell until maximum inhibition was obtained, and then washed off using either the delta antagonist, ICI 174,864 (0.5 μM, R&D) for SNC80-treated cells, or extracellular solution for Baclofen-treated cells. This was followed by the extracellular solution until stable basal currents were obtained.

#### Statistical analysis

Mean Ca^2+^ current amplitudes were measured (pCLAMP 9.0) 5–10 ms after the depolarizing step and basal Ca ^2+^ currents assessed after 4–5 stable recordings were obtained. To control for changes in current amplitude over time, the current amplitude measured before and after drug application was fitted by a linear function to obtain the slope of the basal currents over time. This linear equation was solved for x being the timepoint, or sweep number, at which the drug was applied, so as to obtain the basal current amplitude at the same timepoint as the applied drug. The current amplitude in the presence of the drug was then expressed as a percentage of the basal current amplitude. This was subtracted from 100 to obtain the inhibition in the presence of the drug and expressed as a percentage of the basal current. Data were compared using ANOVA with a posthoc Tukey’s test (Analyse-it-for Microsoft Excel) with significance accepted at p < 0.05 and are expressed as mean ± SEM. Except for recordings that exhibited marked rundown (>30%), all recordings were included in the dataset.

### Inflammatory pain model

All experiments were performed between 8:00–16:00 h. In all cases mice were habituated to the testing area for 20 minutes daily for 2 days prior to baseline testing. For mechanical responses, the threshold for responses to punctate mechanical stimuli (mechanical allodynia) was tested according to the up-and-down method [57]. In this case, the plantar surface of the hindpaw was stimulated with a series of eight von Frey filaments (bending force ranging from 0.01 to 2 g). A response was defined as a lifting or shaking of the paw upon stimulation. Inflammatory pain was induced by injecting Complete Freund’s Adjuvant (CFA, 1 mg *Mycobacterium tuberculosis* (H37Ra, ATCC 25177)/ml of emulsion in 85% paraffin oil and 15% mannide manooleate - Sigma) into the paw. Prior to the injection of CFA baseline mechanical responses (dashed line) were determined. Inflammation was induced by injecting 15 μl of CFA into the plantar surface of the paw, and animals were subsequently tested at different time points post-injection [58]*.* SNC80 was dissolved in 0.9% saline (pH 5.5). SNC80 was administered intraperitoneally in a volume of 10 ml/kg. On the test days (i.e. days 2, 3, 7 and 14 post - CFA) mice were injected with SNC80 or vehicle and tested 45 minutes later. Separate groups of animals were used for days 2, 3 and 7, post-CFA. For day 14, the same group of mice as assayed on day 7 was used.

#### Statistical analysis

For all behavioral experiments data were analyzed using 2-way ANOVA (Sigmastat) and expressed as mean ± SEM.

## Abbreviations

SNC80: ((+)-4-[(α*R*)-α-((2*S*,5*R*)-4-Allyl-2,5-di methyl-1-piperazinyl)-3-methoxybenzyl]-*N*,*N*-diethyl benzamide; CFA: Complete freund’s adjuvant; VDCC: Voltage dependent Ca^2+^ current; δOR: Delta opioid receptor; μOR: Mu opioid receptor

## Competing interests

The authors declare that they have no competing interests.

## Authors’ contributions

AP, MS, and BM carried out the behavioral pain tests and WW performed the electrophysiology experiments. AP, WW and CJE wrote the manuscript. All authors read and approved the final manuscript.
